# TURNOVER AND DEPRESSIVE SYMPTOMS AMONG MEXICAN AMERICAN CAREGIVERS OF PERSONS LIVING WITH DEMENTIA

**DOI:** 10.1093/geroni/igac059.779

**Published:** 2022-12-20

**Authors:** Jacqueline Angel, Sunshine Rote, Kyriakos Markides

**Affiliations:** The University of Texas at Austin, Austin, Texas, United States; University of Louisville, Louisville, Kentucky, United States; UTMB, Galveston, Texas, United States

## Abstract

This study explored the role of caregiver background, stressors, and resources for Mexican American caregiver turnover and depressive symptoms. Using two waves of the Hispanic Established Epidemiologic Study of the Elderly (H-EPESE, 2010/2011-2016 N=333) Caregiver Supplement and informed by the sociocultural caregiver stress process model, we estimate logistic and OLS regressions of change in dementia and change in caregiver over five years. Neuropsychiatric expressions were significantly associated with caregiver turnover. Adult children and grandchildren caregivers were more likely to experience caregiver turnover than spouses. While depressive symptoms were relatively low at both waves, there was a greater increase in depressive symptoms occurred for caregivers who completed the interview in Spanish rather than English, which was partially explained by greater perceived stress at baseline. Findings demonstrate the need to provide dementia care supports for Mexican American caregivers, reduce stress for Spanish-speaking caregivers, and support Mexican American grandchildren who unexpectedly become caregivers.

